# Clinically Applicable System for Rapidly Predicting Enterococcus faecium Susceptibility to Vancomycin

**DOI:** 10.1128/Spectrum.00913-21

**Published:** 2021-11-10

**Authors:** Hsin-Yao Wang, Chia-Ru Chung, Chao-Jung Chen, Ko-Pei Lu, Yi-Ju Tseng, Tzu-Hao Chang, Min-Hsien Wu, Wan-Ting Huang, Ting-Wei Lin, Tsui-Ping Liu, Tzong-Yi Lee, Jorng-Tzong Horng, Jang-Jih Lu

**Affiliations:** a Department of Information Management, National Central University, Taoyuan City, Taiwan; b Ph.D. Program in Biomedical Engineering, Chang Gung University, Taoyuan City, Taiwan; c Department of Computer Science and Information Engineering, National Central University, Taoyuan City, Taiwan; d Graduate Institute of Integrated Medicine, China Medical University, Taichung, Taiwan; e Proteomics Core Laboratory, China Medical University Hospital, Taichung, Taiwan; f Graduate Program in Biomedical Information, Yuan-Ze University, Taoyuan City, Taiwan; g Graduate Institute of Biomedical Informatics, Taipei Medical University, Taipei City, Taiwan; h Clinical Big Data Research Center, Taipei Medical University Hospital, Taipei City, Taiwan; i Graduate Institute of Biomedical Engineering, Chang Gung University, Taoyuan City, Taiwan; j Division of Haematology/Oncology, Department of Internal Medicine, Chang Gung Memorial Hospital at Linkou, Taoyuan City, Taiwan; k Biosensor Group, Biomedical Engineering Research Center, Chang Gung University, Taoyuan City, Taiwan; l School of Life and Health Sciences, The Chinese University of Hong Kong, Shenzhen, China; m Warshel Institute for Computational Biology, The Chinese University of Hong Kong, Shenzhen, China; n Department of Bioinformatics and Medical Engineering, Asia University, Taichung City, Taiwan; o School of Medicine, Chang Gung University, Taoyuan City, Taiwan; p Department of Medical Biotechnology and Laboratory Science, Chang Gung University, Taoyuan City, Taiwan; q Department of Pathology, Kaohsiung Chang Gung Memorial Hospital and Chang Gung University College of Medicine, Kaohsiung, Taiwan; Montefiore Medical Center and Albert Einstein College of Medicine

**Keywords:** vancomycin-resistant *Enterococcus faecium*, antibacterial drug resistance, matrix-assisted laser desorption ionization–time of flight (MALDI-TOF) mass spectrometry, machine learning, rapid detection, *Enterococcus faecium*, clinical methods, microbiology, vancomycin resistance

## Abstract

Enterococcus faecium is a clinically important pathogen that can cause significant morbidity and death. In this study, we aimed to develop a machine learning (ML) algorithm-based rapid susceptibility method to distinguish vancomycin-resistant E. faecium (VRE*fm*) and vancomycin-susceptible E. faecium (VSE*fm*) strains. A predictive model was developed and validated to distinguish VRE*fm* and VSE*fm* strains by analyzing the matrix-assisted laser desorption ionization–time of flight (MALDI-TOF) mass spectrometry (MS) spectra of unique E. faecium isolates from different specimen types. The algorithm used 5,717 mass spectra, including 2,795 VRE*fm* and 2,922 VSE*fm* mass spectra, and was externally validated with 2,280 mass spectra of isolates (1,222 VRE*fm* and 1,058 VSE*fm* strains). A random forest-based algorithm demonstrated overall good classification performances for the isolates from the specimens, with mean accuracy, sensitivity, and specificity of 0.78, 0.79, and 0.77, respectively, with 10-fold cross-validation, timewise validation, and external validation. Furthermore, the algorithm provided rapid results, which would allow susceptibility prediction prior to the availability of phenotypic susceptibility results. In conclusion, an ML algorithm designed using mass spectra obtained from the routine workflow may be able to rapidly differentiate VRE*fm* strains from VSE*fm* strains; however, susceptibility results must be confirmed by routine methods, given the demonstrated performance of the assay.

**IMPORTANCE** A modified binning method was incorporated to cluster MS shifting ions into a set of representative peaks based on a large-scale MS data set of clinical VRE*fm* and VSE*fm* isolates, including 2,795 VRE*fm* and 2,922 VSE*fm* isolates. Predictions with the algorithm were significantly more accurate than empirical antibiotic use, the accuracy of which was 0.50, based on the local epidemiology. The algorithm improved the accuracy of antibiotic administration, compared to empirical antibiotic prescription. An ML algorithm designed using MALDI-TOF MS spectra obtained from the routine workflow accurately differentiated VRE*fm* strains from VSE*fm* strains, especially in blood and sterile body fluid samples, and can be applied to facilitate the rapid and accurate clinical testing of pathogens.

## INTRODUCTION

*Enterococcus* spp. are crucial pathogens in health care-associated infections (1). Enterococcal species can cause a variety of infections, including urinary tract infections and bloodstream infections, and can even result in death (2). Moreover, vancomycin-resistant Enterococcus faecium (VRE*fm*) has placed a heavy burden on health care (3). Early detection of vancomycin resistance is essential for successfully treating VRE*fm* infections (4). The use of vancomycin could be discontinued, and other antibiotics (e.g., linezolid and daptomycin) could be administered, based on the laboratory results of vancomycin resistance (5, 6). Thus, patients’ prognosis could be improved and the development of further drug resistance could be avoided by using antibiotics to which microorganisms are susceptible (5). Moreover, commonly used laboratory drug susceptibility tests, such as the broth microdilution or agar diffusion test, are time-consuming. Antibiotic susceptibility testing (AST) for vancomycin is time-consuming, and the Clinical and Laboratory Standards Institute recommends a full 24 h of incubation for accurate detection of vancomycin resistance in enterococci (7). This could considerably delay timely prescription of antibiotics against E. faecium. Furthermore, prescribing antibiotics based on empirical evidence, without determining AST results, could result in low effectiveness (approximately 50%), depending on the local epidemiology of VRE*fm* (6). Thus, a new scheme is needed to provide rapid and accurate VRE*fm* AST results.

Recently, matrix-assisted laser desorption ionization–time of flight mass spectrometry (MALDI-TOF) mass spectrometry (MS) has become a popular technique among clinical microbiology laboratories worldwide because of its reliability, rapidity, and cost-effectiveness in identifying bacterial species (8–10). In addition to species identification, MALDI-TOF MS has shown potential in other applications, such as strain typing (11, 12) and AST (9, 13). MALDI-TOF MS can generate massive amounts of data with hundreds of peak signals on the spectra (11, 14), the complexity of which can be overwhelming to even an experienced medical staff (12). Some studies have attempted to identify the characteristic peaks by using software protocols (15, 16). However, the approach is typically based on spectrum averages. Peak intensities on MALDI-TOF MS spectra are not quantified, and averaging the spectra is susceptible to biases due to outliers. Furthermore, only a few very discriminative peaks can be identified, and the comprehensive picture for a bacterial characteristic (e.g., antibiotic resistance) may be missed by such an approach. Thus, the results of recent studies have been discordant, which has limited the clinical utility of the tool (17–19).

Machine learning (ML) is a good analytical method for solving classification problems through identification of implicit data patterns from complex data (20). The ML method outperforms traditional statistical methods because of its excellent ability to handle complex interactions among large numbers of predictors and its good performance in nonlinear classification problems. ML has been successfully applied in several clinical fields. Thus, the ML algorithm is especially appropriate for analyzing complex data such as MALDI-TOF MS spectra. To our knowledge, a few studies have used ML in the analysis of MALDI-TOF MS spectra for rapid characterization of VRE*fm*, but the number of cases in those studies were insufficient, limiting the generalization of ML algorithms (21–23). Moreover, none of the studies has comprehensively validated antibiotic susceptibility prediction of ML algorithms by using large real-world data sets to date.

In this study, we aimed to develop and validate a VRE*fm* prediction ML model by using consecutively collected real-world data from two tertiary medical centers (Chang Gung Memorial Hospital [CGMH] Linkou branch and CGMH Kaohsiung branch). Using large real-world MALDI-TOF MS clinical data sets, ML algorithms are expected to predict VRE*fm* more accurately and rapidly and in a ready-to-use manner, which is necessary for clinical applications (24). Moreover, we confirmed the robustness and generalization of the ML algorithm through the following validation methods: cross-validation (CV), timewise internal validations (unseen independent testing data set classified according to time), and external validation (unseen independent testing data set from another medical center). Based on real-world evidence-based validation, our VRE*fm* prediction ML models can be successfully incorporated into routine workflows in clinical laboratories.

## RESULTS

### Predictive peaks for detecting VRE*fm*.

Crucial predictive peaks were defined as those with significantly different frequencies (defined by the chi-square test) in VRE*fm* and vancomycin-susceptible E. faecium (VSE*fm*) samples. We extracted 876 predictor candidates and used a chi-square test to select the important predictive peaks.

**FIG 1:**
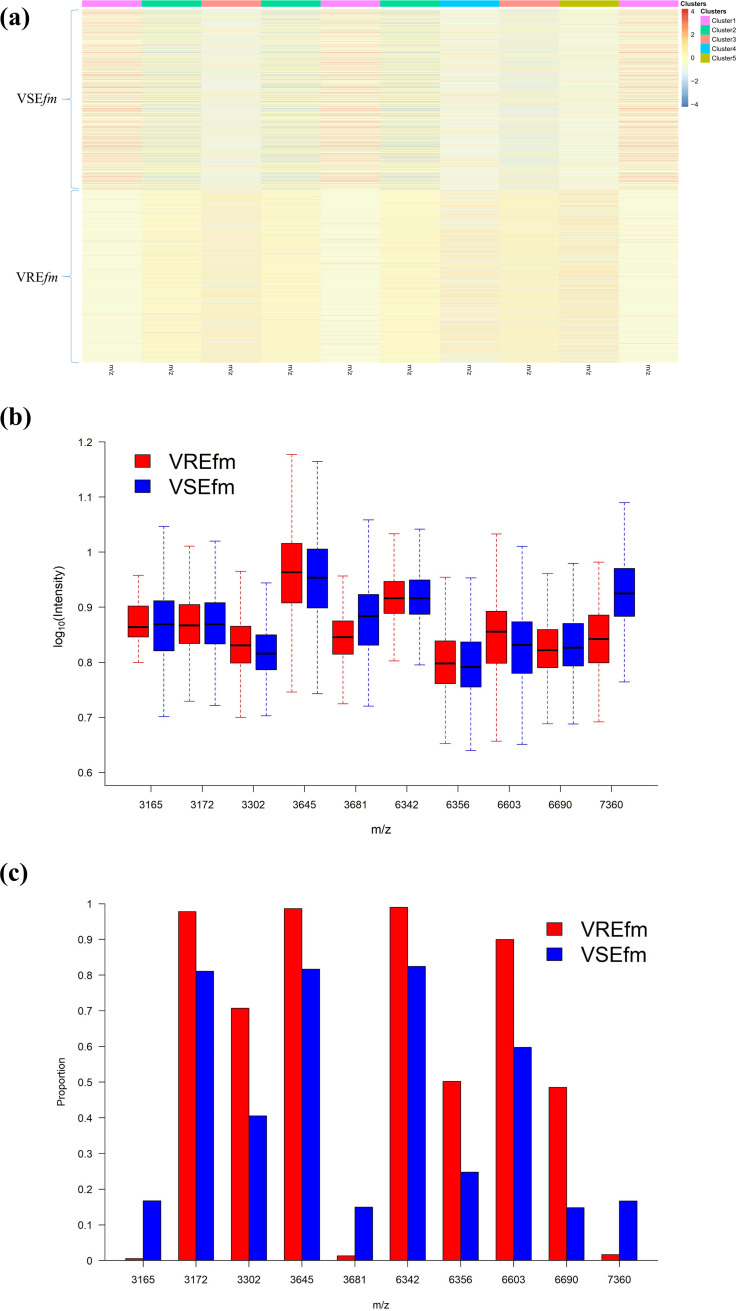
(a) Heat map. We selected the top 10 discriminative peaks by chi-square testing of the occurrence frequency of peaks in VRE*fm* and VSE*fm* (see Table S2 in the supplemental material). The heat map was plotted based on hierarchical clustering of all of the VRE*fm* and VSE*fm* isolates from the CGMH Linkou branch. Rows represent the isolates, and columns represent the top 10 discriminative peaks. The values in the heat map represent the MS spectral intensity, which was log_10_ normalized and Z-score standardized. Red indicates relatively higher peak intensity, while blue indicates lower peak intensity. The isolates are grouped into five clusters. VRE*fm* and VSE*fm* isolates can be visually differentiated by using the top 10 discriminative peaks. (b) Intensity of the top 10 important predictors. The logarithms to base 10 of the peak intensities are plotted for VRE*fm* and VSE*fm*. (c) Occurrence frequency of the top 10 important predictors. The occurrence frequency of the 10 peaks in VRE*fm* and VSE*fm* is plotted.

We selected the 10 most critical predictive peaks and plotted a heat map to visualize the difference between VRE*fm* and VSE*fm* samples (Fig. 1). Peaks of *m/z* 3,172, 3,302, 3,645, 6,342, 6,356, 6,603, and 6,690 were found more frequently in VRE*fm* samples, whereas peaks of *m/z* 3,165, 3,681, and 7,360 occurred more frequently in VSE*fm* samples. Although frequencies for these important predictive peaks were significantly different, we found them in both VRE*fm* and VSE*fm* samples. The full list of crucial predictive peaks is provided in Table S2 in the supplemental material.

We selected several important predictive peaks from the predictor candidate list, which was ordered according to the chi-square scores. Figure S4 in the supplemental material shows the change in performance of ML models when the number of critical predictive peaks was increased. For all of the ML algorithms used in the study, a similar performance trend was observed; the accuracies of the ML models reached a plateau when the number of important predictive peaks included was >100 (see Fig. S4). Thus, the top 100 crucial predictive peaks were selected as the peak composition for the subsequent experiments.

### Performance of VRE*fm* prediction models.

**FIG 2:**
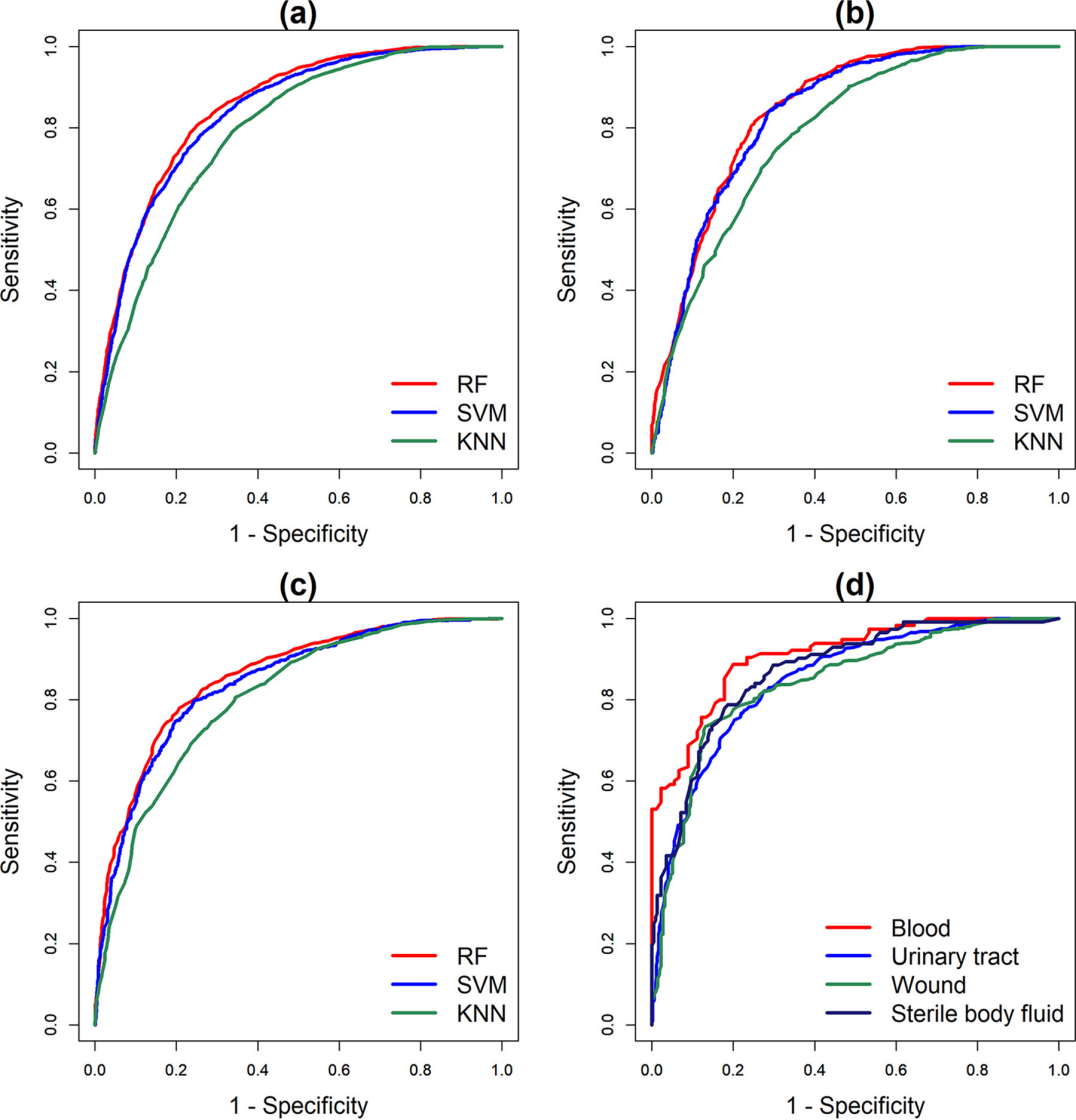
(a) ROC curves for different algorithms in terms of Linkou 5-fold CV. (b) ROC curves for different algorithms in terms of timewise validation. (c) ROC curves for different algorithms in terms of external validation. (d) ROC curves for the RF-based VRE*fm* model with the isolates from different types of specimens.

We summarized the ML models’ performances in Tables 1 and 2 and Fig. 2. The details of the comparisons between different algorithms are described in Table S3. The random forest (RF) model outperformed support vector machine (SVM) and k-nearest neighbor (KNN) in 5-fold CV, timewise internal validation, and external validation (see Table S3); the area under the receiver operating characteristic curve (AUROC) ranged from 0.8463 to 0.8553, and accuracy ranged from 0.7769 to 0.7855. Moreover, performance robustness was also observed with SVM and KNN. Figure 2 shows typical receiver operating characteristic (ROC) curves for the three algorithms in all three validations. We used Youden’s index to select the threshold from the ROC curves in search of balanced sensitivity and specificity. Regarding external validation, the sensitivity and specificity of the RF model were 0.7791 (95% confidence interval [CI], 0.7620 to 0.7961) and 0.7930 (95% CI, 0.7764 to 0.8096), respectively. On the basis of the resistance rate (53.60% VRE*fm*) in the external validation data set, the positive predictive value (PPV) was 0.8130 and the negative predictive value (NPV) was 0.7565.

**TABLE 1:** Performance of VRE*fm* prediction models in terms of k-fold CV, timewise validation, and external validation

Evaluation metrics	Machine learning models:		
	RF model	SVM model	KNN model
AUROC (95% CI)			
5-fold CV	0.8495 (0.8397–0.8594)	0.8367 (0.8264–0.8471)	0.7908 (0.7792–0.8024)
10-fold CV	0.8491 (0.8392–0.8589)	0.8338 (0.8234–0.8442)	0.7589 (0.7468–0.7710)
Timewise validation	0.8463 (0.8273–0.8654)	0.8368 (0.8169–0.8566)	0.7908 (0.7690–0.8127)
External validation	0.8553 (0.8399–0.8706)	0.8407 (0.8246–0.8569)	0.8050 (0.7872–0.8227)
Accuracy (95% CI)			
5-fold CV	0.7769 (0.7660–0.7878)	0.7610 (0.7499–0.7721)	0.7248 (0.7131–0.7364)
10-fold CV	0.7789 (0.7608–0.7827)	0.7587 (0.7476–0.7699)	0.6906 (0.6786–0.7027)
Timewise validation	0.7840 (0.7640–0.8039)	0.7815 (0.7615–0.8016)	0.7228 (0.7011–0.7445)
External validation	0.7855 (0.7687–0.8024)	0.7781 (0.7610–0.7951)	0.7355 (0.7174–0.7536)
Sensitivity (95% CI)			
5-fold CV	0.8054 (0.7951–0.8517)	0.7826 (0.7719–0.7934)	0.7873 (0.7767–0.7980)
10-fold CV	0.7863 (0.7756–0.7969)	0.8192 (0.8091–0.8292)	0.7096 (0.6978–0.7214)
Timewise validation	0.8153 (0.7965–0.8341)	0.8415 (0.8238–0.8592)	0.7491 (0.7281–0.7702)
External validation	0.7791 (0.7620–0.7961)	0.7954 (0.7789–0.8120)	0.8044 (0.7881–0.8207)
Specificity (95% CI)			
5-fold CV	0.7497 (0.7384–0.7609)	0.7403 (0.7289–0.7517)	0.6649 (0.6526–0.6772)
10-fold CV	0.7789 (0.7680–0.7897)	0.7009 (0.6890–0.7128)	0.6725 (0.6603–0.6848)
Timewise validation	0.7477 (0.7266–0.7688)	0.7120 (0.6900–0.7340)	0.6922 (0.6698–0.7146)
External validation	0.7930 (0.7764–0.8096)	0.7580 (0.7405–0.7756)	0.6560 (0.6365–0.6755)

**TABLE 2:** Performance of the RF-based VRE*fm* detection model with different types of specimens in terms of external validation

Metric	Types of specimens:			
	Blood samples (*n* = 205)	Urinary tract samples (*n* = 988)	Sterile body fluid samples (*n* = 338)	Wound samples (*n* = 730)
AUROC (95% CI)	0.9103 (0.8727–0.9480)	0.8494 (0.8258–0.8731)	0.8714 (0.8321–0.9106)	0.8432 (0.8121–0.8743)
Accuracy (95% CI)	0.8488 (0.7997–0.8978)	0.7743 (0.7482–0.8004)	0.8077 (0.7657–0.8497)	0.7740 (0.7436–0.8043)
Sensitivity (95% CI)	0.8870 (0.8436–0.9303)	0.7672 (0.7409–0.7936)	0.7788 (0.7345–0.8230)	0.7339 (0.7018–0.7659)
Specificity (95% CI)	0.8000 (0.7452–0.8548)	0.7805 (0.7547–0.8063)	0.8222 (0.7815–0.8630)	0.8676 (0.8430–0.8922)

Given that the RF algorithm attained the highest performance, we further tested the performance of the RF-based VRE*fm* prediction model using isolates from different types of specimens in the independent testing data set (i.e., external validation by using data from the CGMH Kaohsiung branch) (Table 2). The RF-based VRE*fm* prediction model attained higher performance in predicting VRE*fm* among the isolates from blood and sterile body fluid specimens than from the other specimen types. The AUROC for predicting the isolates from blood specimens reached 0.9103 (95% CI, 0.8727 to 0.9480), whereas that for predicting the isolates from sterile body fluid specimens reached 0.8714 (95% CI, 0.8321 to 0.9106). Moreover, the sensitivity (0.8870 [95% CI, 0.8436 to 0.9303]) and specificity (0.8000 [95% CI, 0.7452 to 0.8548]) in predicting the isolates from blood specimens were also balanced and significantly higher than those for other specimens. In contrast, the performance of the RF-based VRE*fm* prediction model for isolates from urinary tract specimens (0.8494 [95% CI, 0.8258 to 0.8731]) was similar to that for the specimens overall (0.8553 [95% CI, 0.8399 to 0.8706]).

### Protein purification and identification.

**FIG 3:**
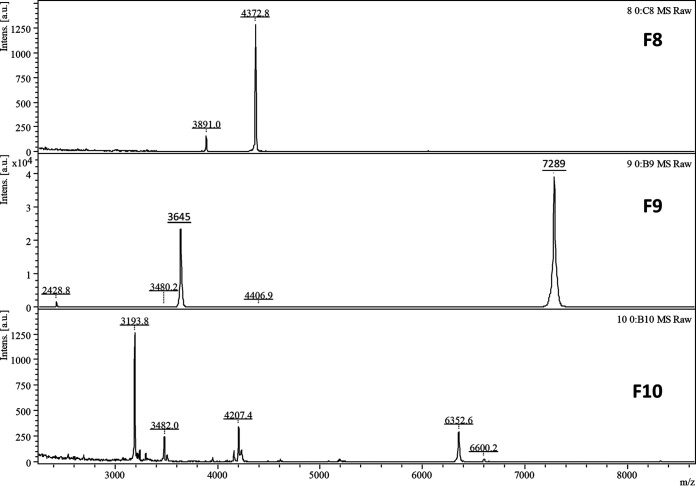
MALD-TOF MS analysis of the C_4_ LC fractions 8 to 10. The peak of *m/z* 3,645 and its singly charged protein peak (*m/z* 7,289) are evident in fraction 9.

**FIG 4:**
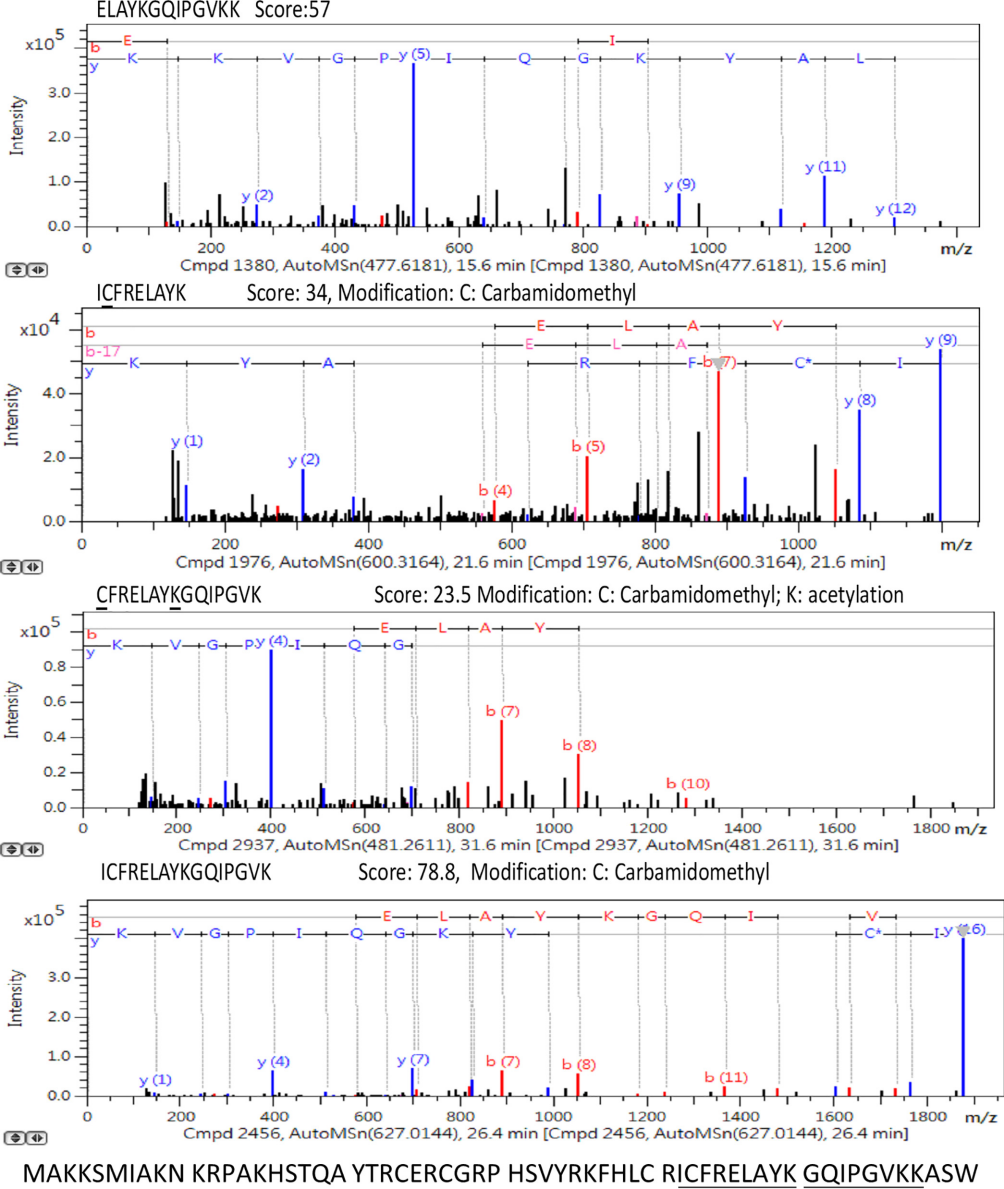
Nano-LC-MS/MS spectra for identification of RS14Z_ENTFA. The identified protein sequence is underlined.

To identify the protein peaks, a C4 liquid chromatography (LC) column was used to purify the peaks, followed by protein digestion and nano-LC-MS/MS analysis. However, even after protein separation on a C4 LC column, it was still challenging to purify a single peptide or protein peak for identification; therefore, only the *m/z* 3,645 peak was identified according to the protein fractionation and nano-LC-MS/MS results. As shown in Fig. 3, after C4 LC column separation, the *m/z* 3,645 ([M+2H]^2+^/2) peak and its singly charged *m/z* 7,289 protein peak were present only in fraction 9. Fractions 8, 9, and 10 were digested and analyzed with nano-LC-MS/MS. We identified the doubly charged *m/z* 3,642 peak (singly charged peak of *m/z* 7,289) as RS14Z_ENTFA (average neutral mass, 7,153 Da), with acetylated modification (Fig. 4).

## DISCUSSION

We developed ML-based models for rapidly and accurately predicting the presence of VRE*fm* in various specimens based on MALDI-TOF MS data. The models revealed higher performance in predicting VRE*fm* in invasive infections (i.e., blood and sterile body fluid samples). We used large-scale real-world data sets to validate the robustness and generalization of the ML-based models, using k-fold CV, timewise internal validation, and external validation. The capability of ML-based models to perform rapid and accurate AST for vancomycin presents a promising breakthrough in determining viable antibiotics against VRE*fm*-related infections.

Our results suggested that antibiotic susceptibility could be predicted accurately by using ML algorithms to analyze MALDI-TOF MS data. MALDI-TOF MS is a powerful analytical tool in current clinical microbiology laboratories, because it offers rapid and cost-effective identification of bacterial species (8–10). Moreover, based on the massive amounts of data produced by MALDI-TOF MS, some studies demonstrated that subspecies typing could be predicted from a specific pattern of MS spectra alone (11, 12). Furthermore, other studies showed good correlation between antibiotic susceptibility and specific patterns of MS spectra (13, 17–19, 25). However, some issues have limited the generalization of these results. First, most of the studies adopted an additional protein extraction step before analytical measurement with MALDI-TOF MS. The protein extraction step could enhance data quality; however, it is not routinely used in clinical practice because it is labor-intensive, time-consuming, and expensive (11, 13). In contrast, we used the direct deposition method, which is recommended by the manufacturer of the instrument and is used for everyday tests. Thus, our models are more feasible for the existing workflow in laboratories because they were adapted using real-world data. Second, the amounts of data in previous studies were too small to be representative. Here, we demonstrated that the ML-based models for predicting VRE*fm* can be applied as a clinical decision support tool using large data sets collected through direct deposition, with various validation methods. Moreover, Idelevich et al. reported that turnaround times could be largely reduced to 5.9 h for MALDI-TOF MS identification purposes (26). Further investigation to determine the suitability of short-term-incubation MALDI-TOF MS spectra for AST would be valuable.

Typically, detection of *vanA* and *vanB* genes is sufficient for reporting the resistance phenotype of enterococcal isolates. However, the nucleic acid test (NAT) is costly and increases economic burdens in health care. Moreover, turnaround times can be long if NATs are performed in a batched manner. Many studies have reported the identification of bacterial susceptibility using infrared spectroscopy with multivariate analysis (27–30). Another study reported that MS in selected-reaction-monitoring mode could be used for the determination of bacterial susceptibility of Staphylococcus aureus to antibiotics in 60 to 80 min, directly from extremely infected patient samples, in contrast to the current time-consuming culture (31). Using either infrared spectroscopy or MS generates multivariate data, and analysis of the complex data with the ML approach could be effective. All approaches explored in the literature report promising data, but case numbers are relatively small. Larger cohorts would be needed to validate the utility and robustness of these promising approaches. In contrast, we used MALDI-TOF MS in tandem with ML algorithms to predict vancomycin resistance in an already identified species (i.e., E. faecium). With this approach, we directly used the MALDI-TOF MS spectra that were generated for species identification, and no additional tests were needed. Thus, our proposed approach would be more cost-effective and clinically practical. Moreover, we demonstrated robustness through internal validation, timewise validation, and external validation. Taken together, these results indicate that the VRE*fm* predictive model is ready to be implemented in clinical settings.

Although identifying crucial predictive peaks for VRE*fm* classification may not be essential in clinical applications, the specific combination of crucial predictive peaks would be beneficial for further studies investigating the molecular mechanism of VRE*fm*. Typically, the *vanA* cluster is the most common mediator of vancomycin resistance in enterococci (32), although many vancomycin resistance genes have been identified (33). Many factors contribute to antibiotic resistance, and the complex mechanisms of antibiotic resistance could evolve in response to the selective pressures of competitive environments (e.g., antibiotic use) (32). Thus, identifying the important predictive peaks for VRE*fm* could help clarify the mechanisms behind resistance. In the present study, for example, *m/z* 6,603, 6,631, and 6,635 peaks were frequent for VRE*fm* (see Table S2 in the supplemental material). These findings are consistent with those obtained in a previous study, which reported that *m/z* 6,603 is specific for *vanB*-positive VRE*fm* strains, while *m/z* 6,631 and 6,635 are specifically found for *vanA*-positive VRE*fm* strains (22). Identification of these peaks needs further investigation, as it could reveal new antibiotics against VRE*fm*.

We purified and identified the peak at *m/z* 3,645, which was one of the most predictive peaks (see Table S2). We identified the doubly charged *m/z* 3,645 peak as 30S ribosomal protein S14 type Z (RS14Z_ENTFA) with acetylated modification. The peak of *m/z* 3,645 (i.e., 30S ribosomal protein S14 type Z) was more frequently identified in VRE*fm* strains than in VSE*fm* strains (Fig. 1c). To the best of our knowledge, the role of 30S ribosomal protein S14 type Z in vancomycin resistance has not yet been reported. Typically, ribosomal proteins are implicated in resistance to macrolides but not glycopeptides (34). Ribosomal protein is stable and hence would be suitable for a diagnostic approach that could be used for the long term. In addition to *m/z* 3,645, our ML-based approach found several potential peaks that were associated with resistance to vancomycin (see Table S2).

Our ML models consistently performed well in 10-fold CV, timewise internal validation, and external validation. Moreover, all of the ML algorithms used in this study exhibited good performances (AUROC values of > 0.8), suggesting that discriminating VRE*fm* from VSE*fm* is generally achievable after adequate feature extraction and selection processes. In timewise internal validation, we intended to simulate a prospective study for a model trained by “past data” to analyze “future data.” Based on their performances in timewise internal validation, we concluded that the trained ML models could also perform well with prospectively collected data, which are not seen in the training process. Results in previous studies differentiating VRE*fm* from VSE*fm* by using MALDI-TOF MS spectra could not be generalized (17–19, 22). Those inconsistent results could be a result of using fewer features (<10). Peak-level reproducibility of MALDI-TOF mass spectra was reported to be approximately 80% (35). Classification performance is compromised when essential peaks are limited and happen to be absent in the mass spectra. The steady and good performance of our ML models could be explained by the inclusion of more than 100 peaks. When some essential peaks were not reproduced in the mass spectra, we used alternative essential peaks for accurate classification. The number of essential peaks indirectly compensated for the insufficient reproducibility of MALDI-TOF MS mass spectra. In predicting VRE*fm* strains from various specimen types, we found that the RF-based model performed especially well with blood and sterile body fluid samples. The superior prediction performance could be attributed to the relatively smaller numbers of VRE*fm* strains in blood and sterile body fluid samples. Bacterial infections in blood or sterile body fluids are typically regarded as invasive infections (36). Only a few VRE*fm* strains (sequence type 17 [ST17], ST18, ST78, and ST203) cause invasive infections in blood or sterile body fluids, according to studies in Taiwan (37) and Ireland (38). The nature of the classification problem would be simpler when the number of labels is fewer.

### Limitations.

This study has several limitations. First, although the models were evaluated using external data from different medical centers, all training data and testing data were collected from only two tertiary medical centers in Taiwan. Therefore, directly applying the ML models in hospitals in other areas or countries, as well as in primary care facilities, may not be feasible. However, we think that the method (but not the trained model) could be generalized. Although our ML models were validated comprehensively using three different approaches and the results showed that the difference in MALDI-TOF MS mass spectra between VRE*fm* strains and VSE*fm* strains could be distinguished with all of the ML algorithms we used, we suggest that others collect locally relevant data for training and validate the VRE*fm* prediction model, given that the epidemiology of VRE*fm* strains could be fairly different, based on sites. Second, our primary goal was to develop and validate a practical and ready-to-use ML model in real-world practice. We found some crucial predictive peaks for VRE*fm*; however, we did not confirm the identities of all of the peaks. The identification of those peaks should be the focus of future investigations. Moreover, the ML model could rapidly predict the vancomycin-susceptible or vancomycin-resistant phenotype for E. faecium strains with clinically useful accuracy; however, we did not test other underlying resistance mechanisms besides *vanA*/*vanB*. Third, we did not use the deep learning (DL) algorithm to predict VRE*fm*, although DL has been successful in the image classification and radiology fields. In this study, VRE*fm* could be accurately predicted using several classic algorithms (i.e., RF, SVM, and KNN), which require fewer resources and less time in training, with the use of models. DL usually requires more training samples and is financially and computationally more expensive than classic ML algorithms (39). DL utility in analyzing MS data rather than image data could be another promising effort in the bioinformatics field. For caregivers, acting on imperfect information is not a revolutionary idea. Compared with the current workflow, the proposed VRE*fm* prediction model would significantly improve antibiotic prescription based on existing MALDI-TOF MS data. Thus, the proposed VRE*fm* prediction model is a better approach than the current workflow and could be clinically useful. To improve the VRE*fm* prediction performance of our models in the clinical setting, clinical information besides MALDI-TOF MS spectra should be included. A more accurate prediction model could be built based on comprehensive clinical and laboratory data.

### Conclusions.

We developed and validated robust ML models capable of discriminating VRE*fm* strains from VSE*fm* strains based on MALDI-TOF MS spectra. These models were especially good at detecting VRE*fm* causing invasive infections. The accurate and rapid detection of VRE*fm* by using the ML models would facilitate more appropriate antibiotic prescription.

## MATERIALS AND METHODS

### Data source.

**FIG 5:**
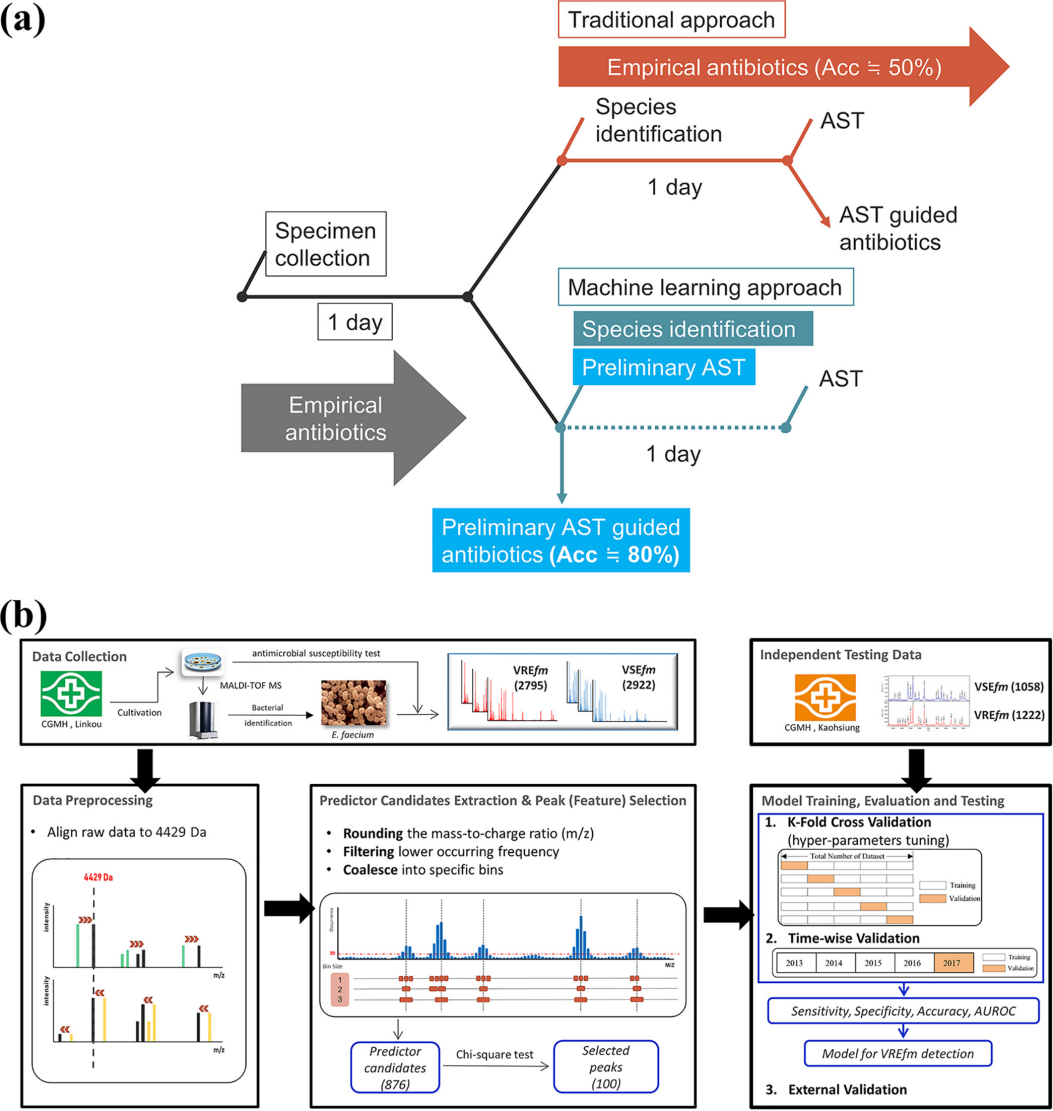
(a) Schematic illustration of the application of the VRE*fm* model. A timeline of the bacterial culture testing using currently used clinical tests (i.e., traditional approach) and a modified timeline with the VRE*fm* model incorporated (i.e., ML approach) are shown. In the traditional approach, specimens are collected for bacterial culture test. Usually, 1 day is needed for growth of a single colony for species identification (by MALDI-TOF MS). Vancomycin AST for VRE*fm* requires another 1 day. In contrast, in the ML approach, the VRE*fm* model can provide preliminary AST results at the time when the bacterial species is identified by MALDI-TOF MS. In the treatment of VRE*fm*, the ML approach can improve the accuracy of antibiotic use. Meanwhile, the turnaround time of the bacterial culture test can be reduced to 1 day, which is a 50% reduction. (b) Schematic illustration of the study design. The study included several steps, i.e., data collection, data preprocessing, predictor candidate extraction and important predictor selection, and model training, evaluation, and testing. Data were obtained from two tertiary medical centers (Linkou and Kaohsiung branches of CGMH). The data included mass spectra and results of the vancomycin susceptibility testing of E. faecium strains. Data from the CGMH Linkou branch were used for model training and validation, while data from the CGMH Kaohsiung branch served as independent testing data. In the steps of data preprocessing and predictor candidate extraction and important predictor selection, a specific set of crucial predictors was used for model training. k-fold, timewise CV, and external validation were used to confirm the models’ robustness. The VRE*fm* prediction model can detect VRE*fm* accurately at least 1 day earlier than the current method can.

We designed a novel ML approach that could improve the accuracy of antibiotic administration and reduce the turnaround time for AST. We summarized the comparison between the ML approach and the traditional approach currently used in clinical microbiology laboratories. The study design is illustrated in Fig. 5. Data used in this retrospective study were consecutively collected from clinical microbiology laboratories at two tertiary medical centers in Taiwan, namely, CGMH Linkou branch and CGMH Kaohsiung branch, between 1 January 2013 and 31 December 2017. The clinical microbiology laboratories of these hospitals collected and processed all routine specimens. In total, 7,997 E. faecium cases were identified and included in this study; of those, 5,717 cases (48.89% VRE*fm*) and 2,280 cases (53.60% VRE*fm*) were obtained from the Linkou and Kaohsiung branches of CGMH, respectively. E. faecium strains were isolated from blood, urinary tract, sterile body fluid, and wound samples. A detailed description of the specimen types is provided in Table S1 in the supplemental material. The study was approved by the institutional review board of Chang Gung Medical Foundation (approval number 201900767B0). We followed the 2015 Standards for Reporting of Diagnostic Accuracy (40) and the Transparent Reporting of a Multivariable Prediction Model for Individual Prognosis or Diagnosis reporting guidelines (41).

### Identification of E. faecium and vancomycin susceptibility.

E. faecium was identified using MALDI-TOF MS spectra measured using a Microflex LT mass spectrometer and analyzed using Biotyper v3.1 (Bruker Daltonik GmbH, Bremen, Germany). A log score (generated through Biotyper v3.1) higher than 2 was considered to confirm the identification of E. faecium (11–13). We tested the vancomycin susceptibility of E. faecium using disk diffusion testing. The details of E. faecium identification and AST are provided in the supplemental material.

### MALDI-TOF MS data collection and preprocessing.

The analytical MALDI-TOF MS measurements were conducted according to the manufacturer’s instructions (Bruker Daltonik GmbH). Single colonies grown on agar were picked and smeared onto a MALDI steel target plate to form thin films. One microliter of 70% formic acid (FA) was applied to the films and dried at room temperature. One microliter of matrix solution (50% acetonitrite (ACN) containing 1% α-cyano-4-hydroxycinnamic acid and 2.5% trifluoroacetic acid) was then added on the films. The sample matrix was dried at room temperature before analysis using a mass spectrometer. MALDI-TOF MS was conducted using a Microflex LT mass spectrometer (Bruker Daltonik GmbH). Mass spectra were obtained under the following settings: linear positive mode; accelerating voltage, +20 kV; nitrogen laser frequency, 60 Hz. In total, 240 laser shots were hit on each sample spot for measurement. The Bruker Daltonics bacterial test standard was used for external calibration of the spectra, and flexAnalysis v3.4 (Bruker Daltonik GmbH) was used for spectrum processing. The Savitzky-Golay algorithm was used for spectrum smoothing. The spectrum baseline was subtracted using the top hat method. The signal-to-noise ratio threshold was set at 2. E. faecium was determined using Biotyper v3.1 (Bruker Daltonik GmbH) on the basis of processed spectra. All of the spectra for the cases reached acceptable quality (log scores of ≥2, as defined by manufacturer’s instruction). Spectra ranging from 2,000 to 20,000 Da were collected for further analysis.

All of the E. faecium isolates had log scores of ≥2 provided by Biotyper v3.1 (Bruker Daltonik GmbH), which ensures the quality of MS spectra (11–13). On this basis, we applied default preprocessing steps, including baseline subtraction, smoothing, and recalibration, to treat the MALDI-TOF MS spectra (range, 2,000 to 20,000 Da) for each isolate by flexAnalysis v3.4 (Bruker Daltonik GmbH) (42). We then extracted the peaks with high occurrence frequency as the predictor candidates through a binning size method developed in a previous study (12, 25), which is illustrated in Fig. S2 in the supplemental material. The extracted peaks were then adjusted according to the alignment of *m/z* 4,429, which was reported to be one of the conserved peaks for E. faecium (15, 43) (see Fig. S3). By alignment to the internal conserved peak, the possible shifting of spectra (12) observed could be adjusted.

### Peak selection from MALDI-TOF MS spectra for model development.

We applied the feature selection method to select the most important peaks from MALDI-TOF MS spectra (44). The peaks were ranked using *P* values for the chi-square test of homogeneity, which was employed to determine whether frequency counts were distributed uniformly across VRE*fm* and VSE*fm* strains. Initially, we selected the top 10 important peaks to plot a heat map based on hierarchical clustering (see the supplemental material). All of the ranked peaks were incorporated in the model accordingly until the performance ceased to improve. Consequently, we obtained important peaks that were highly related to the differentiation of VRE*fm* and VSE*fm* isolates.

To determine the number of peaks included in the ML models, we added them into the ML models and assessed performance using accuracy as the metric. First, the predictor candidates were sorted in a descending order according to the importance score, and one peak was added at a time into the ML models. On the basis of predictive peak composition, we used different algorithms, including RF, SVM with a radial basis function kernel, and KNN, and applied 5-fold CV to the data from the CGMH Linkou branch. The accuracies of the ML models were calculated to determine the adequate number of predictive peaks.

### Development and validation of VRE*fm* prediction models.

The ML-based methods have been successfully applied (in either classification or prediction) in clinical practice (11–13). In this study, three commonly used ML algorithms, namely, RF, SVM with a radial basis function kernel, and KNN, were used to develop the VRE*fm* prediction model. The details of these ML algorithms and model training processes are provided in the supplemental material.

Data from the CGMH Linkou branch were used for 10-fold CV and timewise internal validation; in contrast, data from the CGMH Kaohsiung branch served as the independent testing data for external validation. For 10-fold CV, data were randomly divided into 10 data sets. Each of the 10 data sets served as the testing data set to evaluate the performance of the model developed with the other 9 data sets. In 10-fold CV, we obtained 10 measurements of metrics for evaluating the robustness of the VRE*fm* prediction models. Moreover, to evaluate performance using prospectively collected data, we conducted timewise internal validation; we used data collected between 1 January 2013 and 31 December 2016 as the training data set for developing VRE*fm* prediction models, while data from 1 January 2017 to 31 December 2017 served as the testing data set. To test the generalizability of the models, we used data from the CGMH Linkou branch to develop the predictive models and used data from the CGMH Kaohsiung branch to test the models’ performance in a different medical institution. Additionally, we evaluated the performance of the VRE*fm* prediction model using different types of specimens, such as blood, urine, sterile body fluid, and wound samples, using data from the CGMH Kaohsiung branch. We adopted various metrics, including sensitivity, specificity, accuracy, PPV, NPV, ROC curve, and AUROC, to assess and compare the performance of the VRE*fm* prediction model.

### Purification and digestion of protein markers.

Proteins were extracted from bacterial isolates by adding 100 μl of 70% FA, followed by 100 μl of pure ACN. The extracted protein sample was dried in a centrifugal concentrator (miVac Duo concentrator; Genevac, Stone Ridge, NY, USA) and redissolved in 10 μl of 0.1% FA. For protein subfractionation, 5 μl of protein sample was injected onto a C_4_ LC column (Waters XBridge BEH column [pore size, 300 Å; particle size, 3.5 μm; dimensions, 2.1 by 250 mm]) pumped with a high-performance liquid chromatography (HPLC) system (UltiMate 3000 LC system; Dionex, Amsterdam, Netherlands). Water containing 0.1% (v/v) FA and ACN containing 0.1% (v/v) FA were used as the mobile phase A and mobile phase B, respectively. Gradient elution at a flow rate of 250 μl/min was set as follows: 3 to 30% solvent B for 0 to 8 min, 30 to 70% solvent B for 8 to 16 min, and 70 to 70% solvent B for 16 to 20 min. Protein fractions were collected at 1-min intervals and dried by using a centrifugal concentrator. The collected fractions were redissolved with 0.1% FA and applied to the MALDI-TOF MS system (ultraflex III TOF/TOF; Bruker) to acquire peptide/protein profiling data. The collected protein fraction was then rehydrated with 20 mM ammonium bicarbonate solution, reduced with 10 mM dithiothreitol at 56°C for 15 min, and alkylated with 55 mM iodoacetamide at room temperature for 20 min. Trypsin was added to the protein solution at an enzyme-to-substrate ratio of 1:40 (wt/wt), and the mixture was incubated for 12 h at 37°C for digestion.

### Nano-LC-MS/MS and database search.

Nano-LC-MS/MS was performed using a nanoflow LC system (UltiMate 3000 RSLCnano system; Dionex) coupled to a hybrid quadrupole TOF (Q-TOF) mass spectrometer (maXis impact; Bruker Daltonics). Tryptic peptide mixtures were injected using an autosampler and loaded at a flow rate of 15 μl/min on a self-packed C_18_ trap column for desalting and preconcentration for 5 min. The peptides were then eluted onto an analytical column (Acclaim PepMap C_18_ column [pore size, 100 Å; particle size, 2 μm; dimensions, 75 μm by 250 mm]; Thermo Fisher Scientific, USA) coupled to a nanoelectrospray ionization source on the Q-TOF mass spectrometer. A gradient elution of 1% ACN with 0.1% FA to 40% ACN with 0.1% FA for 90 min was conducted at a flow rate of 300 nl/min for peptide separation. Ten precursors of charge +2, +3, and +4 from each TOF-MS scan were dynamically selected and isolated for MS/MS fragment ion scanning. MS and MS/MS accumulations were set at 1 and 10 Hz, respectively. The MS data were imported into ProteinScape v3.1 (Bruker) and searched in the Swiss-Prot database (release 51.0) using an in-house MASCOT v2.6 server. Search parameters selected were as follows: taxonomy, bacterial; enzyme, trypsin; fixed modifications, carbamidomethyl (C); variable modifications, oxidation (M, H, W) and acetylation (K, N-term); precursor peptide tolerance, 50 ppm; MS/MS tolerance, 0.05 Da; peptide ion score accepted, ≥25.

### Statistical analysis.

The CIs for sensitivity, specificity, and accuracy were estimated using the calculation of the CI for a proportion in one sample situation. Specifically, the critical values followed the Z-score table. To compare the percentages in matched samples, Cochran's *Q* test, a nonparametric approach, was implemented in this study (45). Subsequently, we employed pairwise McNemar's tests (46) for *post hoc* analysis and adopted the false discovery rate proposed by Benjamini and Hochberg to adjust the *P* values (47). Furthermore, the CIs of AUROCs were determined using the nonparametric approach, and the AUROC comparisons mainly adopted the nonparametric approach proposed by DeLong et al. (48).

### Data availability.

The data and codes supporting the conclusions of this report are available from the authors, without undue reservation, to qualified researchers.
