# Erratum for Wang et al., “Clinically Applicable System for Rapidly Predicting Enterococcus faecium Susceptibility to Vancomycin”

**DOI:** 10.1128/spectrum.02610-21

**Published:** 2022-01-19

**Authors:** Hsin-Yao Wang, Chia-Ru Chung, Chao-Jung Chen, Ko-Pei Lu, Yi-Ju Tseng, Tzu-Hao Chang, Min-Hsien Wu, Wan-Ting Huang, Ting-Wei Lin, Tsui-Ping Liu, Tzong-Yi Lee, Jorng-Tzong Horng, Jang-Jih Lu

**Affiliations:** a Department of Laboratory Medicine, Chang Gung Memorial Hospital at Linkou, Taoyuan City, Taiwan; b Ph.D. Program in Biomedical Engineering, Chang Gung University, Taoyuan City, Taiwan; c Department of Computer Science and Information Engineering, National Central University, Taoyuan City, Taiwan; d Graduate Institute of Integrated Medicine, China Medical University, Taichung, Taiwan; e Proteomics Core Laboratory, China Medical University Hospital, Taichung, Taiwan; f Graduate Program in Biomedical Information, Yuan-Ze University, Taoyuan City, Taiwan; g Department of Information Management, Chang Gung University, Taoyuan City, Taiwan; h Graduate Institute of Biomedical Informatics, Taipei Medical University, Taipei City, Taiwan; i Clinical Big Data Research Center, Taipei Medical University Hospital, Taipei City, Taiwan; j Graduate Institute of Biomedical Engineering, Chang Gung University, Taoyuan City, Taiwan; k Division of Haematology/Oncology, Department of Internal Medicine, Chang Gung Memorial Hospital at Linkou, Taoyuan City, Taiwan; l Biosensor Group, Biomedical Engineering Research Center, Chang Gung University, Taoyuan City, Taiwan; m Department of Pathology, Kaohsiung Chang Gung Memorial Hospital and Chang Gung University College of Medicine, Kaohsiung, Taiwan; n School of Life and Health Sciences, The Chinese University of Hong Kong, Shenzhen, China; o Warshel Institute for Computational Biology, The Chinese University of Hong Kong, Shenzhen, China; p Department of Bioinformatics and Medical Engineering, Asia University, Taichung City, Taiwan; q School of Medicine, Chang Gung University, Taoyuan City, Taiwan; r Department of Medical Biotechnology and Laboratory Science, Chang Gung University, Taoyuan City, Taiwan

## ERRATUM

Volume 9, no. 3, e00913-21, 2021, https://doi.org/10.1128/Spectrum.00913-21. Page 1: The affiliations should appear as given in this Erratum.

Page 13, Acknowledgments, line 3: “CMRPG3L0401, CMRPG3L0431” should be added after “CMRPG3J1791.”

