# Supplementary material for: Clinically Applicable System for Rapidly Predicting Enterococcus faecium Susceptibility to Vancomycin
Source: Microbiol Spectr. 2021 Nov 10;9(3):e00913-21. doi: 10.1128/Spectrum.00913-21 (PMC8579932; doi:10.1128/Spectrum.00913-21)
Supplement: SUPPLEMENTAL FILE 1 — Supplemental material. Download SPECTRUM00913-21_Supp_1_seq9.pdf, PDF file, 1.5 MB [file spectrum00913-21_supp_1_seq9.pdf]

Supporting Information

A clinically applicable system for rapidly detecting vancomycin-resistant *Enterococcus faecium* strains using MALDI-TOF MS spectra obtained routinely from real-world microbiology laboratories of hospitals

Hsin-Yao Wang[1,2], Chia-Ru Chung[3], Chao-Jung Chen[4,5], Ko-Pei Lu[6], Yi-Ju Tseng[1,7], Tzu-Hao Chang[8,9], Min-Hsien Wu[1,9,10,11,12], Wan-Ting Huang[18], Ting-Wei Lin[1], Tsui-Ping Liu[1], Tzong-Yi Lee[13,14*], Jorng-Tzong Horng[1,3,15*], Jang-Jih Lu[1,16,17*]

[1]Department of Laboratory Medicine, Chang Gung Memorial Hospital at Linkou, Taoyuan City, [2]Ph.D. Program in Biomedical Engineering, Chang Gung University, Taoyuan City, Taiwan, [3]Department of Computer Science and Information Engineering, National Central University, Taoyuan City, Taiwan, [4]Graduate Institute of Integrated Medicine, China Medical university, Taichung, Taiwan, [5]Proteomics Core Laboratory, China Medical University Hospital, Taichung, Taiwan, [6]Graduate Program in Biomedical Information, Yuan-Ze University, Taoyuan City, Taiwan, [7]Department of Information Management, Chang Gung University, Taoyuan City, Taiwan, [8]Graduate Institute of Biomedical Informatics, Taipei Medical University, Taipei City, Taiwan, [9]Clinical Big Data Research Center, Taipei Medical University Hospital, Taipei City, Taiwan, [10]Graduate Institute of Biomedical Engineering, Chang Gung University, Taoyuan City, Taiwan, [11]Division of Haematology/Oncology, Department of Internal Medicine, Chang Gung Memorial Hospital at Linkou, Taoyuan City, Taiwan, [12]Biosensor Group, Biomedical Engineering Research Center, Chang Gung University, Taoyuan City, Taiwan, [13]School of Life and Health Sciences, The Chinese University of Hong Kong, Shenzhen, China, [14]Warshel Institute for Computational Biology, The Chinese University of Hong Kong, Shenzhen, China, [15]Department of Bioinformatics and Medical Engineering, Asia University, Taichung City, Taiwan, [16]School of Medicine, Chang Gung University, Taoyuan City, Taiwan, [17]Department of Medical Biotechnology and Laboratory Science, Chang Gung University, Taoyuan City, Taiwan, [18]Department of Pathology, Kaohsiung Chang Gung Memorial Hospital and Chang Gung University College of Medicine, Kaohsiung, Taiwan.

*To whom correspondence should be addressed:
Tzong-Yi Lee, PhD
School of Life and Health Sciences, The Chinese University of Hong Kong, Shenzhen 518172, China
Warshel Institute for Computational Biology, The Chinese University of Hong Kong, Shenzhen 518172, China
Jorng-Tzong Horng, PhD
Department of Computer Science and Information Engineering, National Central University, Taoyuan 32001, Taiwan
Department of Bioinformatics and Medical Engineering, Asia University, Taichung 41359, Taiwan
Jang-Jih Lu, M.D., Ph.D.
Department of Laboratory Medicine, Chang Gung Memorial Hospital at Linkou
Department of Medical Biotechnology and Laboratory Science, Chang Gung University
5 Fu-Shing St. Kweishan
Taoyuan 333, Taiwan

Supplemental Methods

Specimen processing, *Enterococcus faecium* identification, and vancomycin susceptibility test

Clinical specimens were continuously collected as daily routine from all the wards to the clinical microbiology laboratory of Chang Gung Memorial Hospital, both Linkou and Kaohsiung branches. The specimen types included blood, respiratory tract specimen (*ie,* sputum, bronchial wash, and bronchoalveolar lavage), sterile cavity fluid (*ie*, ascites, pleural effusion, pericardial effusion, cerebrospinal fluid, and synovial fluid), tip of implant, urine, wound, and others. The distribution of specimens is summarized in Supplementary Table 1. Blood specimens were collected after aseptic preparation and cultured in trypticase soy broth (Becton Dickinson, MD, USA). Positive culture results were detected using the automated detection system (BD BACTEC™ FX; Becton Dickinson). Blood was drawn out from positive blood culture bottles onto blood plate (BP) agar for subculture (Becton Dickinson, MD, USA). Sputum specimens with adequate quality[1] were used. The respiratory specimens were inoculated on BP agar (Becton Dickinson), eosin methylene blue (EMB) agar (Becton Dickinson), CNA agar (Becton Dickinson), and chocolate agar (Becton Dickinson). Specimens obtained from the sterile cavity fluid were inoculated on BP, EMB, CNA, and chocolate agars, and into thioglycollate broth (Becton Dickinson). While positive growth was noted in thioglycollate broth, subculture was performed using BP agar. A semiquantitative culture method described by Maki et al. was used for testing the tip of implants.[2] Urine specimens were inoculated using a quantitative loop on BP and EMB agars. For specimens collected from wound, 1.2 mL of 0.9% saline was used for rinsing when the specimens were obtained using a swab. The rinsed saline was inoculated on BP, EMB, CNA, and chocolate agars; for pus collected from wound, the specimens were directly dropped on the agars and into thioglycollate broth. The agar and broth were incubated in a CO_2 incubator at 37°C for 18–24 hours. Single colonies grown on agar plates were picked for further analysis. *Enterococcus faecium* was identified based on colony morphology and matrix-assisted laser desorption ionization time-of-flight (MALDI-TOF) spectra (Bruker Daltonik GmbH, Bremen, Germany). The paper disc method was used to differentiate vancomycin-resistant *Enterococcus* from vancomycin-susceptible *Enterococcus* on the basis of Clinical and Laboratory Standards Institute guidelines M100. The susceptibility of vancomycin was interpreted according to CLSI M100 of the corresponding years. The interpretative criteria of vancomycin for *E. faecium* do not change in the period of 2013-2017.

Supplementary Table 1. Specimen Distribution

**Total *E. faecium* Linkou (5717)**	**Blood (674)**	**Urinary tract (2818)**	**Sterile body fluid (1009)**	**Wound (1211)**	**Respiratory tract(3)**	**Others (2)**
S	263(39.0%)	1343(47.7%)	664(65.8%)	651(53.8%)	1(33.3%)	0(0%)
R	411(61.0%)	1475(52.3%)	341(34.2%)	560(46.2%)	2(66.7%)	2(100%)
**Total *E. faecium* Kaohsiung (2280)**	**Blood (205)**	**Urinary tract (988)**	**Sterile body fluid (338)**	**Wound (730)**	**Respiratory tract (1)**	**Others (18)**
S	90(43.9%)	524(53.0%)	225(66.6%)	219(30.0%)	0(0%)	0(0%)
R	115(56.1%)	464(47.0%)	113(33.4%)	511(70.0%)	1(100%)	18(100%)

We only selected specimens of sufficient quantity, that is, blood, urinary tract, sterile body fluid, and wound, for building the vancomycin-resistant *E. faecium* (VRE*fm*) prediction model in this study.

Binning method for extracting predictor candidates

In a MALDI-TOF mass spectrometry (MS) spectra, the peaks were extracted and regarded as predictors for the construction of predictive models. In an initial scanning through all MS spectra, a peptide would locate at subtle different m/z location in multiple replications. The range of peaks drifting was reported ± 5 m/z[4,7]. The shifting/drifting problem of peaks is illustrated in Supplementary Figure 1. To deal with this problem among different spectra, the binning method was adopted to group large-scale peaks into a smaller number of "bins". Supplementary Figure 2 presents a schematic

diagram of the binning method used in this study. The peaks located within the same bin are considered as the same feature. In the binning method, we evaluated various bin sizes (0-12 Da), and we adopted 10 Da as the bin size for the following experiments given the results of evaluation and the previous studies.[4,7] Moreover, on the basis of binning results, we aligned the peaks according to *m/z* 4429 to obtain more accurate *m/z* positions for the peaks. The peptide at *m/z* 4429 was reported to be fairly abundant in *E. faecium*.[8,9] Thus, we selected the peptide at *m/z* 4429 as the intrinsic internal control to adjust the other peaks (Supplementary Figure 3). In the study, we could find peaks that are close to the m/z 4429 across all *E. faecium* isolates.

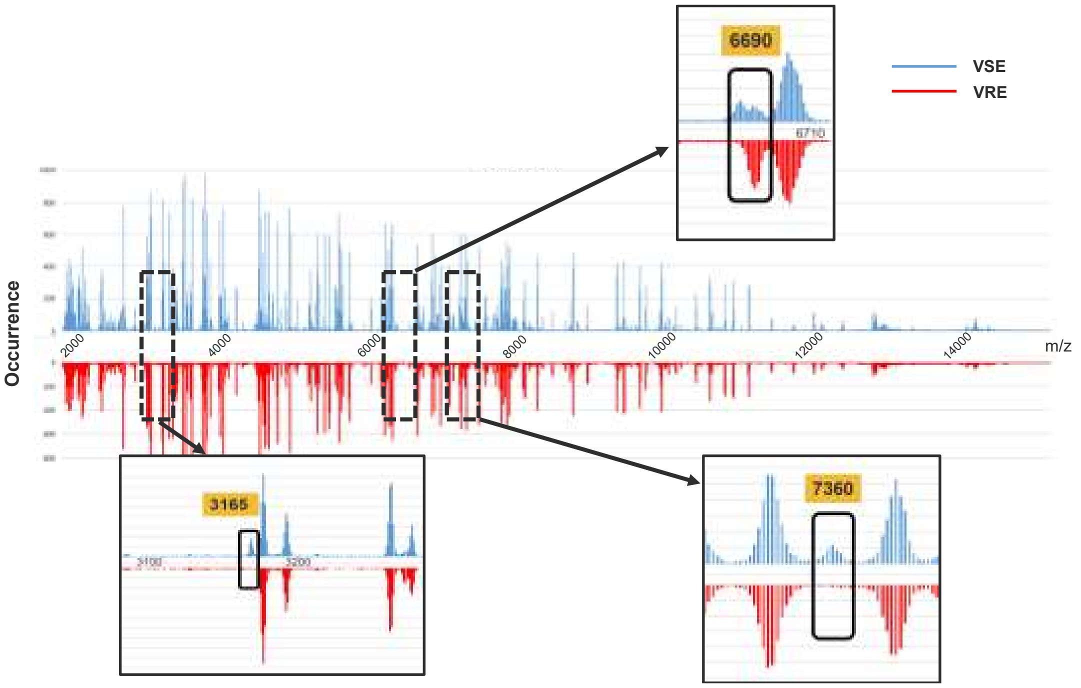

Supplementary Figure 1. An Example of MALDI-TOF MS Spectra to Illustrate the "Shifting Problem". Peptides of the same species do not appear at exactly the same *m/z* position. By contrast, the peptides of the same species would distribute in a normal distribution-like manner.

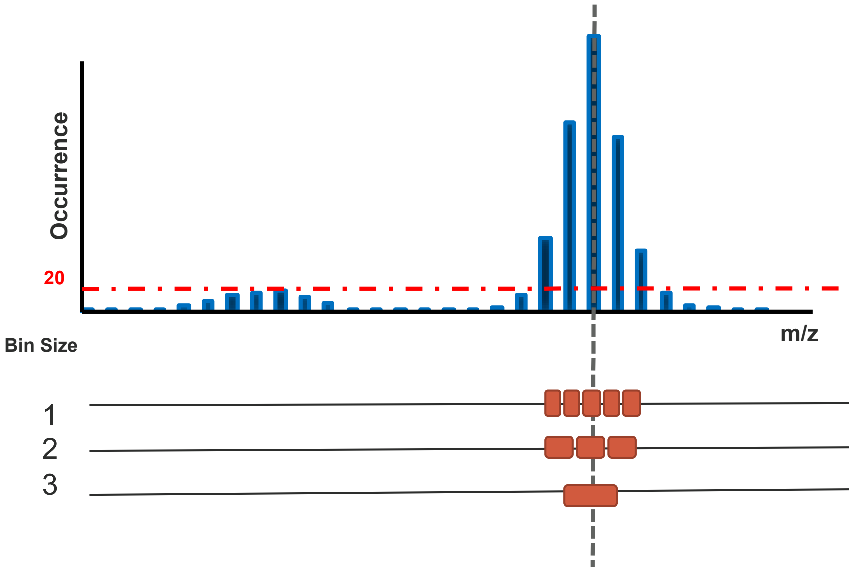

Supplementary Figure 2. Schematic Illustration of the Binning Method. In the original MS spectra, the peptides of the same species were located at several different *m/z* positions nearby. We

used the binning method to classify these peaks into smaller groups. An example is illustrated: Occurrence frequency at a specific *m/z* larger than 20% of all cases is taken into calculation. When the bin size is 1, then 5 resulting features are obtained; when the bin size is 3, only 1 resulting feature is obtained.

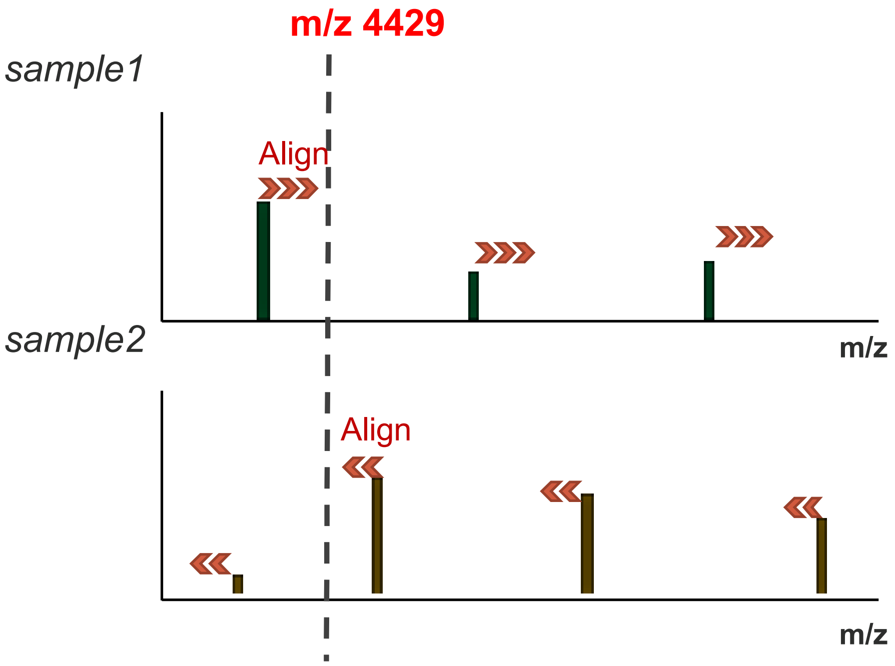

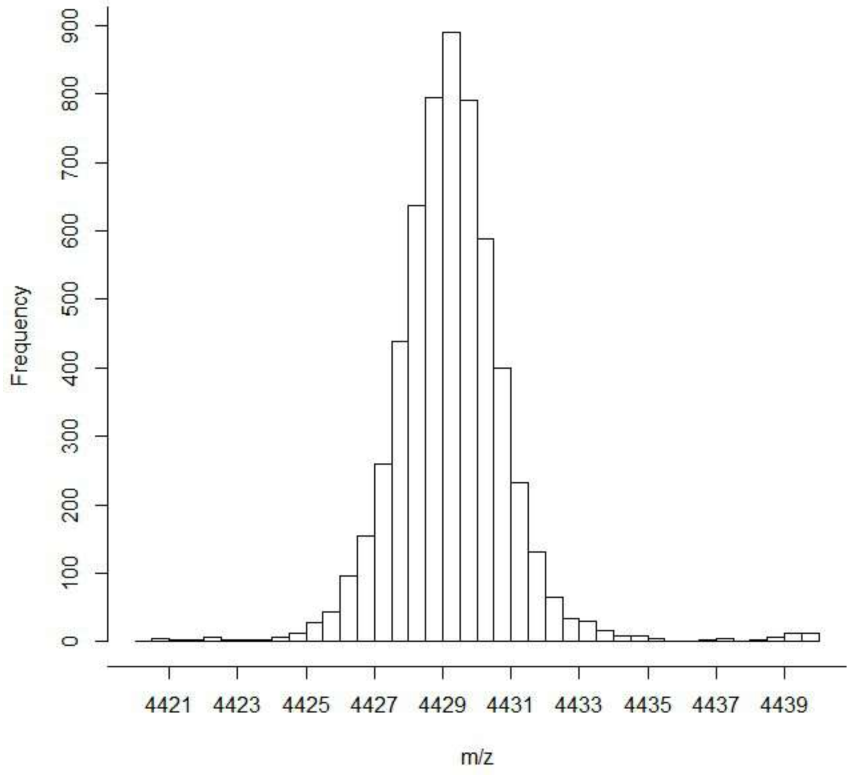

Supplementary Figure 3. (a) Examples of alignment to *m/z* 4429. We illustrated 2 examples of alignment: The upper one shows shifting to the right when the peak located closest to *m/z* 4229 is shifted from the left to right, and then, the other peaks on the spectra also shifted to the right. By contrast, the lower figure shows a case of shifting to the left when the peak

closest to *m/z* 4429 shifted from right to left. (b) Peaks distribution around m/z 4429 for all the isolates. Based on the histogram, most of the isolates have peaks located in m/z 4429 ± 5. Thus, most of the MS spectra were shifted less than 5 m/z.

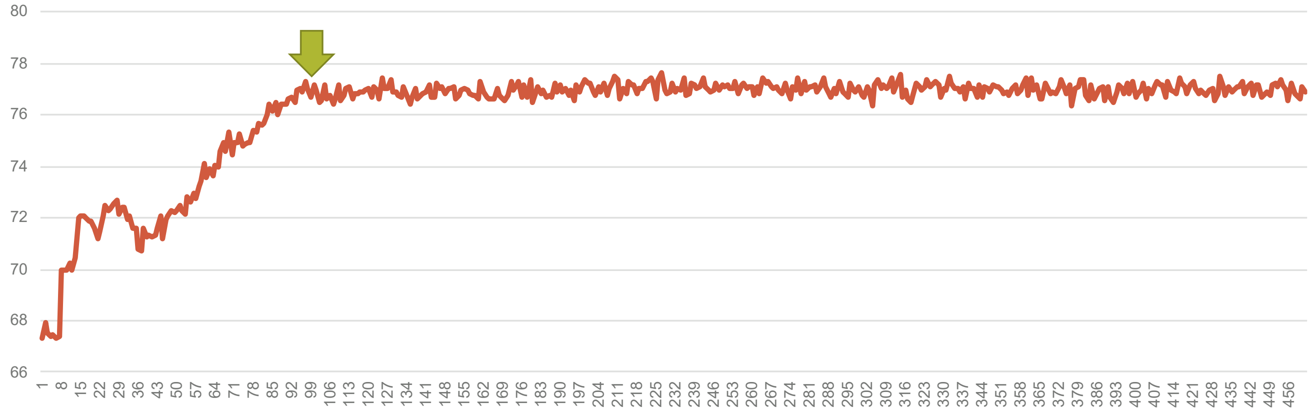

Supplementary Figure 4. Performance of ML Models Based on Different Number of Predictors. The performance reaches a plateau when the number of predictors is more than 100.

Heat map

On the basis of the chi-square scores of the predictor candidates (ie, peaks), we selected top 10 most critical predictive peaks (Supplementary Table 2) and plotted a heat map using hierarchical clustering. We took log of the intensity of the top 10 most critical predictive peaks, followed by z-score standardization. The hierarchical clustering was conducted based on Euclidean distance metric and average linkage. We produced the heat map by using pheatmap package on R software (version 3. 3. 3, R Foundation for Statistical Computing, http://www.r-project.org/).

Random forest

Random forest (RF), an ensemble learning method, is widely used for classification. More specifically, the ensemble learning approach combines multiple learning models to obtain an improved classification model and thus obtain better performance on prediction.[10] Additionally, the bootstrap aggregation (bagging) technique is considered for sampling the training data in RF. In other words, the bootstrap method samples the training tuples averagely with replacement, which means every selected tuple is likely to be re-added to the training set. In this study, we adopted the Weka toolkit[11] to construct RF classifiers based on various feature sets.

K-Nearest Neighbors

The nearest neighbor approach is an instance-based classifier used for determining the most similar instances, which were selected from all training data, to a given test instance, based on a distance function. Given a test instance, the most *k* similar instances are regarded as *k*-nearest neighbors (KNNs) of the test data, and the class assignment is determined in accordance with the proportion of KNNs. Considering the training data and test data as the *n*-dimensional vectors in Gaussian space, the Euclidean distance function is usually applied to measure the distances between the test data and all training data. Given a test instance *t*, the Euclidean distance between *t* and a training instance *x* is defined as

$$\mathbf{d}(\boldsymbol{t},\boldsymbol{x}) = \sqrt{\sum_{\boldsymbol{k}=1}^{\boldsymbol{n}} (\boldsymbol{t_k} - \boldsymbol{x_k})^2}$$

where *n* is the size of the feature set. After determining KNNs, the class labels of these k training instances might be inconsistent. A weighted distance voting method was used to conduct the class assignment for a test data. Class assignment C(*t*) of a test data *t* is determined by

$$\mathbf{C}(\boldsymbol{t}) = \underset{v}{\mathbf{argmax}} \sum_{x_i \in KNNs} \boldsymbol{w_i} \times \boldsymbol{I}(\boldsymbol{x_i}) \qquad \begin{cases} \boldsymbol{I}(\boldsymbol{x_i}) = \mathbf{1} \;\; \boldsymbol{if} \;\; \boldsymbol{v} = \boldsymbol{class \;\; label \;\; of \;\; x_i} \\ \boldsymbol{I}(\boldsymbol{x_i}) = \mathbf{0} \;\; \boldsymbol{if} \;\; \boldsymbol{v} \neq \boldsymbol{class \;\; label \;\; of \;\; x_i} \end{cases}$$

where *v* is the class label and $\boldsymbol{w_i}$ is the weighted value of the class label of $\boldsymbol{x_i}$ in KNNs. For a binary classification between VRE*fm* and vancomycin-susceptible *E. faecium* (VSE*fm*) samples, the positive and negative training instances were represented as *n*-dimensional vectors with class labels +1 and −1, respectively. The testing data without class labels are classified into +1 or −1 based on the *k* nearest training samples. In the KNN classifier, various values of *k* were examined to find the best cutoff with best performance.

Support Vector Machine

This study involved a binary classification of VRE*fm* and VSE*fm* spectra. The positive (VRE*fm*) and negative (VSE*fm*) spectra were labeled as +1 and −1, respectively, for the 2 classes. The training dataset is $X = \{x^t, c^t\}$ where $c^t = +1$ if $x^t \in$ positive dataset and $c^t = -1$ if $x^t \in$ negative dataset. This study attempted to identify *w* and w_0 such that

$w^T x^t + w_0 \geq +1 \quad for \;\; c^t = +1$ and $w^T x^t + w_0 \leq -1 \quad for \;\; c^t = -1$,

which can be rewritten as

$$c^t(w^T x^t + w_0) \geq +1$$

This formula could be used to estimate the optimal separating hyperplane that can maximize the margin between 2 classes.[12] The distance of x^t to the discriminating hyperplane is

$$\frac{|w^T x^t + w_0|}{||w||}$$

and we would like the distance to be higher than a specific value *h*:

$$\frac{c^t(w^T x^t + w_0)}{||w||} \geq h, \forall t \;\; and \;\; c^t \in \{+1, -1\}$$

The support vector machine (SVM) is an advanced algorithm used to identify a hyperplane between 2 classes with a maximum margin based on an *n*-dimensional vector space.[12] In an attempt to maximize *h*, however, an unlimited number of possible values could be elucidated by tuning *w*. Hence, $h||w||$ was defined as one and $||w||$ was minimized using the following equation[13]:

$$min \frac{1}{2} ||w||^2 \;\; subject \;\; to \;\; c^t(w^T x^t + w_0) \geq +1, \forall t$$

In this work, SVM could be adopted to determine a hyperplane for discriminating between VRE*fm* and VSE*fm* samples with a maximal margin in a vector space containing *n* dimensions (size of the feature set). The mass-to-charge ratio values of spectra were represented as a numeric vector in an n-dimensional vector space, which are the input values for SVM. A famous SVM public resource, called LIBSVM,[14] was downloaded and installed in our computing server for an iterative training of multiple SVMs in accordance with various feature sets. With ML, if the best discriminant is nonlinear, instead of enabling a nonlinear model, we could map all *n*-dimensional vectors to a new vector space with higher dimension *m*, where *m* > *n*, based on nonlinear kernel functions. As demonstrated in previous methods,[15-19] the radial basis function (RBF) has been typically chosen as the specified kernel function in SVM models. The RBF function was given as follows:

$$K(x^t, x) = \exp\left[-\frac{||x^t - x||^2}{2s^2}\right]$$

where x^t is the center and *s* is the radius, which should be provided by the programmer. With LIBSVM, cost (*c*) and gamma (*r*) are 2 supporting parameters used to optimize the radius of the kernel function and softness of the hyperplane, respectively. To achieve the feasible values of gamma (*r*) and cost (*c*) in model learning, an optimization program, written in Python, was provided by LIBSVM.

Supplementary Table 2. Top 100 Important Predictors Calculated Using Chi-Square

Predictor (m/z)	X-squared	P-value
6690	749.6721	4.73E-165
6603	680.4819	5.25E-150
3302	520.7866	2.85E-115
3165	461.9951	1.77E-102
6342	451.5601	3.30E-100
3645	450.855	4.70E-100
3172	412.4703	1.06E-91
6356	391.5542	3.80E-87
7360	375.9846	9.32E-84
3681	344.9242	5.40E-77
7289	317.5502	4.95E-71
3655	299.2888	4.71E-67
6661	271.7419	4.73E-61
6528	254.4583	2.77E-57
6512	246.2366	1.72E-55
12711	233.6196	9.68E-53
3740	211.9791	5.08E-48
7306	208.538	2.86E-47
6741	199.5891	2.57E-45
3651	197.2695	8.24E-45
3900	175.462	4.75E-40
6361	174.2589	8.69E-40
10625	167.6077	2.46E-38
3870	164.2027	1.37E-37
5949	145.6891	1.52E-33
6328	137.147	1.12E-31
13482	133.0305	8.90E-31
3264	130.0682	3.96E-30
6631	126.649	2.22E-29
7385	122.6643	1.65E-28
7310	120.8941	4.03E-28
3884	110.0463	9.57E-26
3306	99.5212	1.94E-23
6748	94.5692	2.37E-22
10629	90.9076	1.51E-21
2430	86.3685	1.49E-20
12715	84.3901	4.06E-20
5935	77.9294	1.07E-18
6665	76.8498	1.84E-18
7413	68.7908	1.09E-16

3906	68.5522	1.24E-16
2720	68.3585	1.36E-16
3316	60.3783	7.83E-15
6308	59.4367	1.26E-14
3256	59.0654	1.53E-14
6333	58.9954	1.58E-14
5478	52.5241	4.25E-13
12720	50.6321	1.11E-12
3875	50.3945	1.26E-12
4857	48.9752	2.59E-12
7743	48.5973	3.14E-12
12724	48.5585	3.21E-12
7364	48.0801	4.09E-12
6607	45.4681	1.55E-11
6635	41.967	9.28E-11
7753	41.6587	1.09E-10
5768	40.7215	1.76E-10
3518	40.3004	2.18E-10
5313	39.0606	4.11E-10
6493	38.438	5.65E-10
4831	37.1236	1.11E-09
2967	37.0867	1.13E-09
6460	36.16	1.82E-09
9714	34.0623	5.34E-09
4573	33.8151	6.06E-09
3694	32.8717	9.84E-09
6477	32.6426	1.11E-08
7034	32.3444	1.29E-08
7418	31.5142	1.98E-08
4469	31.0475	2.52E-08
3013	30.8891	2.73E-08
3915	30.3285	3.65E-08
6752	30.3066	3.69E-08
9773	30.2625	3.77E-08
10957	29.5373	5.49E-08
9953	29.1471	6.71E-08
13385	29.1427	6.72E-08
7330	29.0184	7.17E-08
10941	28.9438	7.45E-08
7445	28.6333	8.75E-08
3337	28.5984	8.91E-08
3321	27.3422	1.70E-07

5038	25.7023	3.98E-07
3724	25.4256	4.60E-07
5220	25.0366	5.63E-07
12728	24.7339	6.58E-07
4861	24.3722	7.94E-07
7275	24.3091	8.20E-07
5387	23.9425	9.93E-07
10952	23.9008	1.01E-06
5485	23.6539	1.15E-06
6346	23.6241	1.17E-06
4844	23.5285	1.23E-06
6105	23.485	1.26E-06
5383	23.3773	1.33E-06
10075	22.8121	1.79E-06
8031	22.4537	2.15E-06
2449	22.0113	2.71E-06
6818	21.9906	2.74E-06
3027	21.2967	3.93E-06

Supplementary Table 3(a). Comparison of AUROCs Between Different Algorithms With Different Validation Methods

	5-fold CV	Time-wise internal validation	External validation
KNN-RF	1.37×10^{-45}	4.94×10^{-11}	1.24×10^{-15}
KNN-SVM	7.16×10^{-24}	6.49×10^{-7}	2.49×10^{-7}
RF-SVM	3.33×10^{-8}	0.0310	0.0001

Table 3(b). Comparison of Accuracies Between Different Algorithms With Different Validation Methods, and *P* Values of Cochran's Q Test

Datasets	RF	SVM	KNN	p-value
5-fold CV	0.7769 (0.7660, 0.7878)	0.7610 (0.7499, 0.7721)	0.7248 (0.7131, 0.7364)	2.20×10^{-16}
Time-wise internal validation	0.7840 (0.7640, 0.8039)	0.7815 (0.7615, 0.8016)	0.7228 (0.7011, 0.7445)	1.72×10^{-12}
External validation	0.7855 (0.7687, 0.8024)	0.7781 (0.7610, 0.7951)	0.7355 (0.7174, 0.7536)	5.15×10^{-11}

Table 3(c). Comparison of Sensitivities Between Different Algorithms With Different Validation Methods, and *P* Values of Cochran's Q Test

Datasets	RF	SVM	KNN	p-value
5-fold CV	0.8054 (0.7951, 0.8517)	0.7826 (0.7719, 0.7934)	0.7873 (0.7767, 0.7980)	0.0038
Time-wise internal validation	0.8153 (0.7965, 0.8341)	0.8415 (0.8238, 0.8592)	0.7491 (0.7281, 0.7702)	1.50×10^{-12}
External validation	0.7791 (0.7620, 0.7961)	0.7954 (0.7789, 0.8120)	0.8044 (0.7881, 0.8207)	0.0265

Table 3(d). Comparison of Specificities Between Different Algorithms With Different Validation Methods, and *P* Values of Cochran's Q Test

Datasets	RF	SVM	KNN	p-value
5-fold CV	0.7497 (0.7384, 0.7609)	0.7403 (0.7289, 0.7517)	0.6649 (0.6526, 0.6772)	2.20×10^{-16}
Time-wise internal validation	0.7477 (0.7266, 0.7688)	0.7120 (0.6900, 0.7340)	0.6922 (0.6698, 0.7146)	0.0002
External validation	0.7930 (0.7764, 0.8096)	0.7580 (0.7405, 0.7756)	0.6560 (0.6365, 0.6755)	2.20×10^{-16}

Table 3(e). Pairwise McNemar's Test for Sensitivity, Specificity, and Accuracy Between Different Algorithms With Different Validation Methods

Comparison	Adjusted p-value of comparing two sensitivities	Adjusted p-value of comparing two specificities	Adjusted p-value of comparing two accuracies
(a) 5-fold CV			
KNN-RF	0.0284	1.08×10^{-26}	7.86×10^{-21}
KNN-SVM	0.5640	5.58×10^{-20}	5.62×10^{-10}
RF-SVM	8.70×10^{-5}	0.0909	4.14×10^{-5}
(b) Time-wise internal validation			

KNN-RF	4.44×10^{-6}	4.28×10^{-4}	1.14×10^{-8}
KNN-SVM	1.10×10^{-9}	0.2100	7.46×10^{-8}
RF-SVM	3.76×10^{-3}	3.45×10^{-4}	0.7050
(c) External validation			
KNN-RF	0.0366	1.25×10^{-21}	1.36×10^{-8}
KNN-SVM	0.3830	3.00×10^{-13}	8.20×10^{-7}
RF-SVM	0.0618	3.94×10^{-4}	0.2350

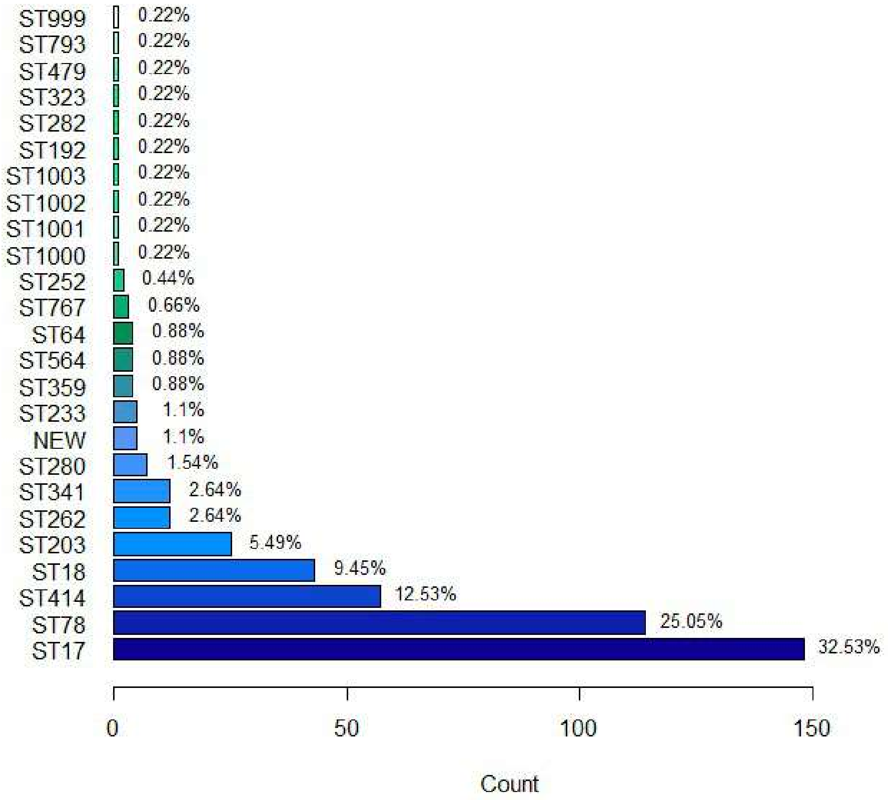

Supplementary Figure 5. **Distribution of the sequence type (ST) for the VRE*fm* isolates.**

Supplementary Table 4. Implement of the VRE*fm* predictive model. VA*: predicted susceptibility test by the ML model.

No	Time to Prediction	Time to Final	Time saved	VA	VA*
1	41	64	23	S	S
2	18	119	101	R	R
3	21	49	28	R	R
4	30	75	45	R	R
5	31	75	44	S	S
6	30	50	20	R	R
7	23	71	48	R	R
8	20	71	51	R	R
9	20	43	23	S	S
10	38	157	119	R	R
11	27	49	22	R	R
12	31	53	22	S	S
13	40	85	45	R	R
14	37	59	22	R	R
15	27	94	67	R	R
16	27	76	49	R	R
17	23	68	45	R	R
18	29	78	49	R	R
19	19	93	74	R	R
20	20	92	72	R	R
21	22	68	46	R	R
22	25	48	23	R	R
23	20	57	37	R	R
24	28	56	28	R	R
25	14	42	28	R	R
26	25	48	23	S	S
27	38	85	47	R	R
28	23	48	25	R	R
29	33	57	24	R	R
30	14	70	56	R	R
31	19	67	48	R	R
32	34	64	30	R	R
33	38	63	25	S	S
34	38	63	25	S	S
35	19	42	23	S	S
36	20	46	26	R	R
37	23	73	50	R	R
38	26	49	23	R	R

39	25	49	24	R	R
40	25	47	22	S	S
41	22	67	45	R	R
42	47	70	23	R	R
43	39	107	68	R	R
44	20	48	28	S	S
45	18	50	32	R	R
46	24	49	25	S	S
47	63	90	27	S	S
48	25	73	48	R	R
49	25	51	26	R	R
50	23	47	24	R	R
51	22	48	26	R	R
52	22	47	25	R	R
53	27	95	68	R	R
54	28	95	67	R	R
55	38	62	24	S	S
56	35	62	27	S	S
57	35	62	27	S	S
58	18	68	50	R	R
59	22	67	45	S	S
60	26	46	20	S	S
61	29	99	70	R	R
62	68	117	49	R	R
63	28	50	22	R	R
64	28	50	22	R	R
65	28	48	20	R	R
66	21	42	21	R	R
67	21	42	21	R	R
68	42	73	31	R	R
69	37	88	51	R	R
70	29	71	42	R	R
71	16	89	73	R	R
72	29	98	69	R	R
73	19	67	48	R	R
74	19	67	48	S	S
75	38	62	24	R	R
76	31	54	23	R	R
77	25	44	19	R	R
78	23	45	22	S	S
79	37	61	24	R	R

80	26	72	46	R	R
81	39	108	69	R	R
82	26	51	25	S	S
83	19	61	42	R	R
84	24	48	24	S	S
85	20	64	44	R	R
86	41	84	43	R	S
87	28	95	67	R	S
88	23	46	23	S	R
89	62	134	72	R	S
90	46	69	23	S	R
91	17	61	44	S	R
92	43	70	27	S	R
93	25	97	72	R	S
94	23	43	20	S	R
95	18	39	21	R	S
96	26	48	22	R	S
97	26	51	25	S	R
98	22	67	45	S	R
99	38	83	45	R	S
100	25	46	21	S	R
101	21	50	29	S	R
102	22	93	71	R	S
103	42	94	52	S	R
104	23	50	27	S	R
105	16	44	28	S	R
106	22	65	43	R	S
107	30	73	43	S	R

Reference:

1. Murray PR, Washington JA. Microscopic and baceriologic analysis of expectorated sputum. *Mayo Clin Proc.* 1975;50(6):339-344.
2. Maki DG, Weise CE, Sarafin HW. A semiquantitative culture method for identifying intravenous-catheter-related infection. *N Engl J Med.* 1977;296(23):1305-1309.
3. Wang H-Y, Lien F, Liu T-P, Chen C-H, Chen C-J, Lu J-J. Application of a MALDI-TOF analysis platform (ClinProTools) for rapid and preliminary report of MRSA sequence types in Taiwan. *PeerJ.* 2018;6.
4. Wang HY, Lee TY, Tseng YJ, et al. A new scheme for strain typing of methicillin-resistant Staphylococcus aureus on the basis of matrix-assisted laser desorption ionization time-of-flight mass spectrometry by using machine learning approach. *PLoS One.* 2018;13(3):e0194289.
5. Wang HY, Chen CH, Lee TY, et al. Rapid Detection of Heterogeneous Vancomycin-Intermediate Staphylococcus aureus Based on Matrix-Assisted Laser Desorption Ionization Time-of-Flight: Using a Machine Learning Approach and Unbiased Validation. *Front Microbiol.* 2018;9:2393.
6. Camoez M, Sierra JM, Dominguez MA, Ferrer-Navarro M, Vila J, Roca I. Automated categorization of methicillin-resistant Staphylococcus aureus clinical isolates into different clonal complexes by MALDI-TOF mass spectrometry. *Clin Microbiol Infect.* 2016;22(2):161 e161-161 e167.
7. Mather CA, Werth BJ, Sivagnanam S, SenGupta DJ, Butler-Wu SM. Rapid Detection of Vancomycin-Intermediate Staphylococcus aureus by Matrix-Assisted Laser Desorption Ionization-Time of Flight Mass Spectrometry. *J Clin Microbiol.* 2016;54(4):883-890.
8. Stepien-Pysniak D, Hauschild T, Rozanski P, Marek A. MALDI-TOF Mass Spectrometry as a Useful Tool for Identification of Enterococcus spp. from Wild Birds and Differentiation of Closely Related Species. *J Microbiol Biotechnol.* 2017;27(6):1128-1137.
9. Lasch P, Fleige C, Stämmler M, et al. Insufficient discriminatory power of MALDI-TOF mass spectrometry for typing of Enterococcus faecium and Staphylococcus aureus isolates. *Journal of microbiological methods.* 2014;100:58-69.
10. Liang SY, Wu SW, Pu TH, Chang FY, Khoo KH. An adaptive workflow coupled with Random Forest algorithm to identify intact N-glycopeptides detected from mass spectrometry. *Bioinformatics.* 2014;30(13):1908-1916.
11. Hall M, Frank E, Holmes G, Pfahringer B, Reutemann P, Witten IH. The WEKA data mining software. *ACM SIGKDD Explorations Newsletter.* 2009;11(1).
12. Vapnik VN. An overview of statistical learning theory. *IEEE Trans Neural Netw.* 1999;10(5):988-999.
13. Byvatov E, Schneider G. Support vector machine applications in bioinformatics. *Appl Bioinformatics.* 2003;2(2):67-77.
14. Chang C-C, Lin C-J. Libsvm. *ACM Transactions on Intelligent Systems and Technology.* 2011;2(3):1-27.
15. Kumari B, Kumar R, Kumar M. PalmPred: an SVM based palmitoylation prediction method using sequence profile information. *PLoS One.* 2014;9(2):e89246.

16. Lu CT, Chen SA, Bretana NA, Cheng TH, Lee TY. Carboxylator: incorporating solvent-accessible surface area for identifying protein carboxylation sites. *J Comput Aided Mol Des.* 2011;25(10):987-995.
17. Lee TY, Chen SA, Hung HY, Ou YY. Incorporating distant sequence features and radial basis function networks to identify ubiquitin conjugation sites. *PLoS One.* 2011;6(3):e17331.
18. Chang WC, Lee TY, Shien DM, et al. Incorporating support vector machine for identifying protein tyrosine sulfation sites. *J Comput Chem.* 2009;30(15):2526-2537.
19. Wong YH, Lee TY, Liang HK, et al. KinasePhos 2.0: a web server for identifying protein kinase-specific phosphorylation sites based on sequences and coupling patterns. *Nucleic Acids Res.* 2007;35(Web Server issue):W588-594.
